# Gingerol suppresses sepsis-induced acute kidney injury by modulating methylsulfonylmethane and dimethylamine production

**DOI:** 10.1038/s41598-018-30522-6

**Published:** 2018-08-14

**Authors:** Francisco Adelvane de Paulo Rodrigues, Alan Diego da Conceição Santos, Pedro Henrique Quintela Soares de Medeiros, Mara de Moura Gondim Prata, Tailane Caína de Souza Santos, James Almada da Silva, Gerly Anne de Castro Brito, Armênio Aguiar dos Santos, Edilberto Rocha Silveira, Aldo Ângelo Moreira Lima, Alexandre Havt

**Affiliations:** 1 Department of Physiology and Pharmacology, School of Medicine, Federal University of Ceará, Fortaleza, CE Brazil; 2 Department of Organic and Inorganic Chemistry, Federal University of Ceará, Fortaleza, CE Brazil; 3 Department of Pharmacy, Federal University of Sergipe, Lagarto, SE Brazil

## Abstract

Acute kidney injury (AKI) and metabolic dysfunction are critical complications in sepsis syndrome; however, their pathophysiological mechanisms remain poorly understood. Therefore, we evaluated whether the pharmacological properties of 6-gingerol (6G) and 10-gingerol (10G) could modulate AKI and metabolic disruption in a rat model of sepsis (faecal peritonitis). Animals from the sham and AKI groups were intraperitoneally injected with 6G or 10G (25 mg/kg). Septic AKI decreased creatinine clearance and renal antioxidant activity, but enhanced oxidative stress and the renal mRNA levels of tumour necrosis factor-α, interleukin-1β, and transforming growth factor-β. Both phenol compounds repaired kidney function through antioxidant activity related to decreased oxidative/nitrosative stress and proinflammatory cytokines. Metabolomics analysis indicated different metabolic profiles for the sham surgery group, caecal ligation and puncture model alone group, and sepsis groups treated with gingerols. ^1^H nuclear magnetic resonance analysis detected important increases in urinary creatine, allantoin, and dimethylglycine levels in septic rats. However, dimethylamine and methylsulfonylmethane metabolites were more frequently detected in septic animals treated with 6G or 10G, and were associated with increased survival of septic animals. Gingerols attenuated septic AKI by decreasing renal disturbances, oxidative stress, and inflammatory response through a mechanism possibly correlated with increased production of dimethylamine and methylsulfonylmethane.

## Introduction

Patients with sepsis usually develop acute kidney injury (AKI), with an incidence reaching 23–51% (severe sepsis to septic shock)^1^. The mortality rates related to septic and non-septic AKI are 70% and 45%, respectively^2^. With regard to functional parameters, septic AKI is characterized by decreased glomerular filtration rate (GFR), increased blood urea nitrogen (BUN) level, electrolyte/acid-base disorder, and fluid overload^3,4^. This phenotype seems to be dependent on the overproduction of reactive oxygen species (ROS) and reactive nitrogen species (RNS), which can cause capillary disturbances, glomerular hypoperfusion, tubular dysfunction, and renal hypoxia^3,5^.

In addition, systemic and local production of inflammatory biomarkers plays a critical role in the evolution of kidney disorders in patients with systemic inflammatory response syndrome (SIRS)^6^. Renal synthesis of interleukin (IL)−1β, tumour necrosis factor (TNF)-α, interferon (IFN)-γ, and other cytokines triggers injury of the tubular endothelium, which is associated with worsening of renal function after an infective insult^6,7^.

Metabolic disruption is another serious and alarmingly frequent process in the pathophysiology of sepsis and septic shock^8^. Septic AKI associated with metabolic disruption has a multifactorial pathogenesis and is related to longer hospital stays, higher burden of comorbidities, worse outcome, and limited therapy^2,9^. Metabolic characterization and understanding its consequences are of great interest and clinically necessary^10^. Conversely, recent studies on metabolic sepsis have provided interesting insights into this phenomenon^11,12^.

Studies that simultaneously evaluated AKI and metabolic disruption in patients with sepsis are scarce. Therefore, we investigated the effects of the phenol compounds 6-gingerol (6G) and 10-gingerol (10G) on this critical pathogenic event. These bioactive molecules from ginger (*Zingiber officinale* Rosc.) have important antioxidant, anti-inflammatory^13^, immunomodulatory, and metabolic activities^14^. Previously, our group reported that a gingerol-enriched fraction containing 6G and 10G attenuated the ROS activation and mRNA expression of proinflammatory cytokines (IL-2, IL-β, TNF-α, INF-γ) in an aminoglycoside-induced nephrotoxicity model^15^. *In vivo* studies have indicated beneficial activities of ginger compounds against kidney disorders^16^, such as relief of acute tubular necrosis (ATN)^17^ and ROS scavenging in renal cells^18^. The mechanisms involved in the nephroprotection and metabolic improvement induced by 6G and 10G treatments remain undefined. We hypothesized that these phenolic compounds could protect the renal function against sepsis. Thus, we investigated the effects of 6G and 10G in a caecal ligation and puncture (CLP)–induced septic AKI model, by evaluating the oxidative status and anti-inflammatory response, as well as the metabolomic profile of the animals.

## Results

### Ginger compounds ameliorated renal function and showed anti-oxidative activity

Animals subjected to the CLP procedure showed deterioration of kidney function indicated by oliguria and decreased creatinine clearance (CrCl), followed by increases in urinary protein, BUN, and serum creatinine (SCr) levels; urinary osmolality; and sodium fraction excretion (FE_NA_). These results are clear signs of AKI. Both gingerols protect the kidneys from the negative effects of sepsis (*P* < *0*.*01*) (Table 1).

**Table 1:** Pathophysiological parameters in sepsis-induced AKI.

Variable	Value (mean SEM) for group					
	Sham	Sham-6G	Sham-10G	CLP	CLP + 6G	CLP + 10G
UV (mL/24 h)	22.5 ± 2.8	21.33 ± 1.8	24.08 ± 2.2	11.98 ± 1.6*	22.70 ± 2.7^#^	23.33 ± 2.3^#^
U_Osm_ (mOsm/kg)	362.4 ± 66.3	368.1 ± 72.9	288.4 ± 16.0	496.1 ± 52.97*	320.9 ± 45.64	287.9 ± 25.37^#^
FE_NA_ (%)	0.43 ± 0.07	0.45 ± 0.05	0.67 ± 0.15	1.2 ± 0.19*	0.61 ± 0.14^#^	0.58 ± 0.09^#^
SCr (mg/dL)	0.37 ± 0.03	0.41 ± 0.03	0.40 ± 0.03	0.75 ± 0.05**	0.48 ± 0.03^#^	0.48 ± 0.3^#^
CrCl (ml/min)	1.45 ± 0.2	1.28 ± 0.2	1.27 ± 0.1	0.46 ± 0.1*	1.23 ± 0.22^##^	1.02 ± 0.07^#^
Urea (mg/dL)	45.6 ± 4.2	44.75 ± 3.7	39.38 ± 3.4	62.14 ± 2.1**	46.83 ± 2.7^#^	60.50 ± 1.1
Urinary prot. (mg/dL)	12.37 ± 3.1	11.29 ± 1.8	11.34 ± 1.8	46.67 ± 7.4*	22.95 ± 7.1^#^	21.58 ± 4.2^#^

The urine volume (UV); urinary osmolality (U_OSM_); sodium fractional excretion (FE_NA_), creatinine (SCr), creatinine clearance (CrCl), BUN and urinary protein were different significantly in septic AKI compared to sham-operated group. Gingerol intervention ameliorated these physiological responses. The results are expressed as means ± SEM. Statistical analysis was performed by ANOVA followed by Bonferroni’s multiple comparison test (n = 6–8). **and *denote statistical significance compared to the sham group (*P* < *0*.*01*, *P* < *0*.*05*, respectively); ^#^and ^##^show significant difference compared to CLP group (*P* < *0*.*01*, *P* < *0*.*05*, respectively).

**Figure 1:**
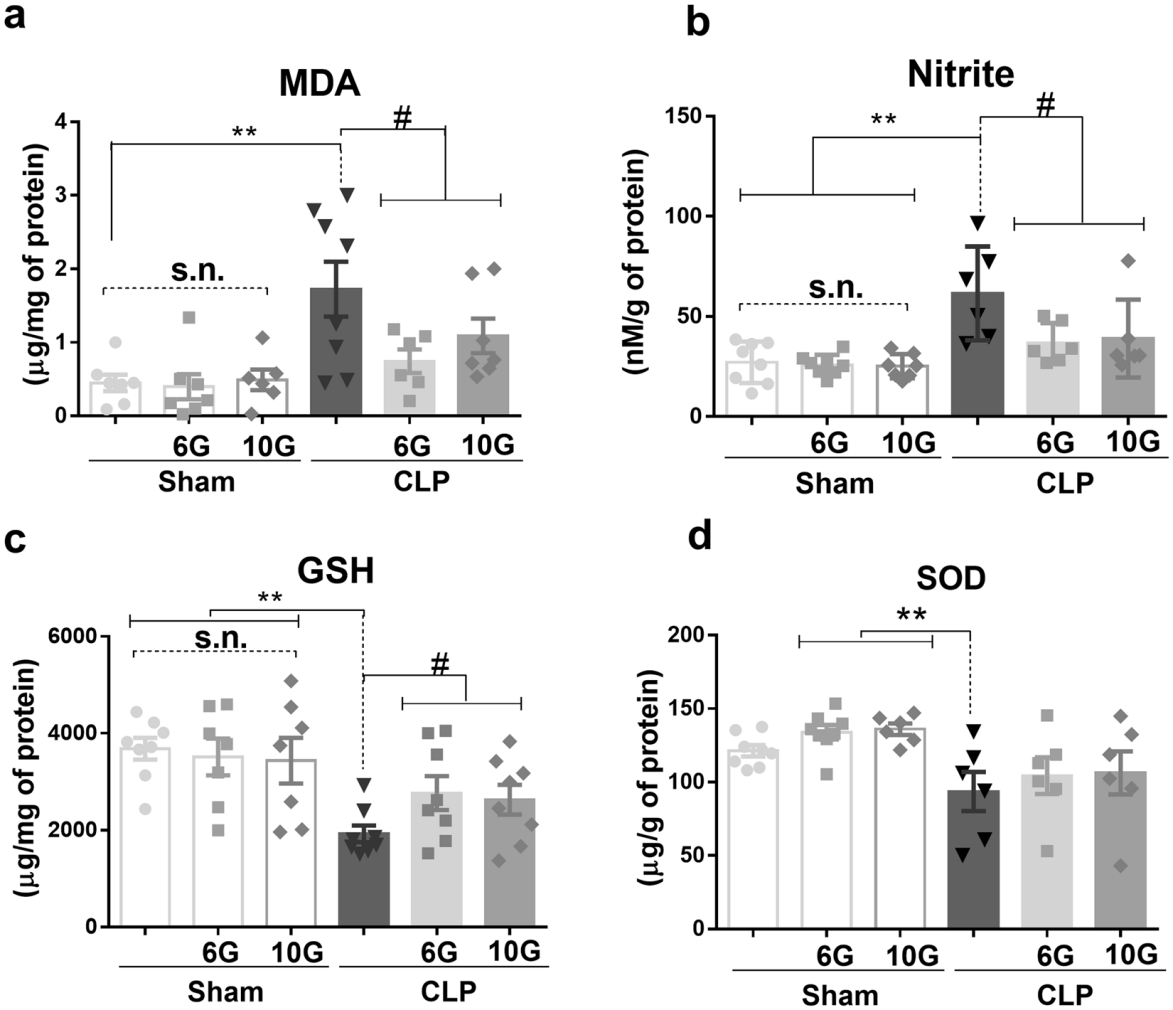
Kidney redox profile. (**a**) Oxidative activities showed by the increased amounts of malondialdehide (MDA), (**b**) nitrite level, (**c**) but also by the reduced activity of glutathione (GSH) and (**a**) superoxide dismutase (SOD), indicating functional and cell disturbance in CLP model. On the other hand, gingerols showed antioxidant activity. The results are expressed as means ± SEM (n = 7–8). Statistical analysis was performed by ANOVA followed by Bonferroni’s multiple comparison test. **and *denote statistical significance compared to the sham group (*P* < *0*.*01*, *P* < *0*.*05*, respectively); ^#^and ^##^show significant difference compared to CLP group (*P* < *0*.*01*, *P* < *0*.*05*, respectively).

Sepsis augmented the oxidative and nitrosative stress in the kidney, indicated by higher values of malondialdehyde and nitrite levels, respectively. In addition, the levels of reduced glutathione (GSH) and superoxide dismutase (SOD), which are enzymes protective against cell stress, decreased. Both ginger compounds showed anti-oxidative and anti-nitrosative activities that also ameliorated the GSH content (*P* < *0*.*05*) (Fig. 1a–d).

### Gingerols reduced the renal mRNA expression of inflammatory, tubular injury, and immune biomarkers, and improved the survival rate

**Figure 2:**
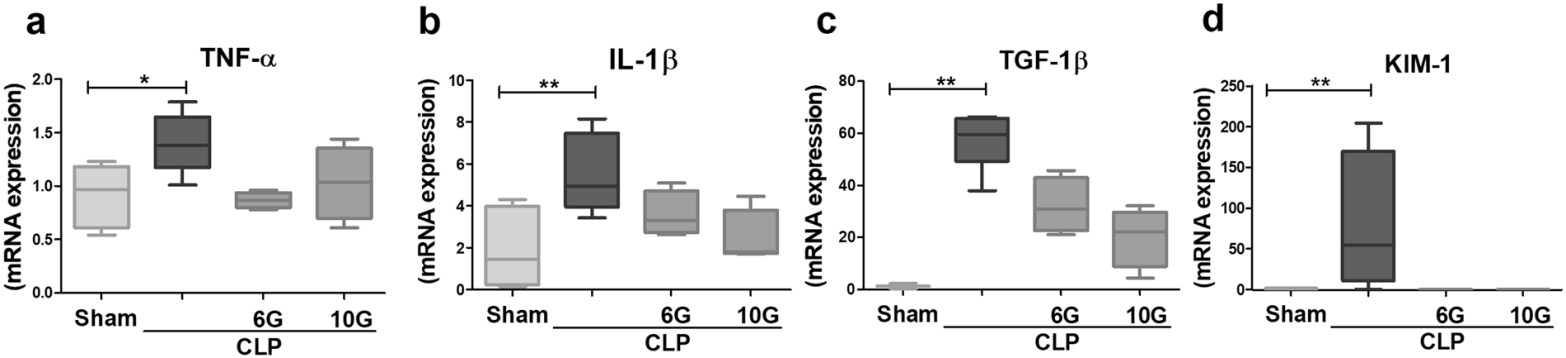
Gingerols suppress the mRNA expression of proinflammatory cytokines and renal biomarkers. CLP animals increased in kidney tissue the quantitative relative expression of (**a**) TNF-α, (**b**) IL-1β, (**C**) TGF-β1 and (**d**) KIM-1 mRNAs in the sepsis-induced AKI. Treatment with 6G and 10G decreased the pro-inflammatory cytokine of animals induced to septic AKI. Statistical analysis was performed by the Mann-Whitney test for RT-qPCR (n = 5–6). **and *denote statistical significance compared to the sham group (*P* < *0*.*05*, *P* < *0*.*01*, respectively); ^#^show significant difference compared to CLP group (*P* < *0*.*05*).

We also analysed the mRNA expression of TNF-α, IL-1β, transforming growth factor-β1 (TGF-β1), and kidney injury molecule-1 (KIM-1) in renal tissue samples obtained from the CLP alone and CLP + gingerol treatment groups. Sepsis increased the mRNA expression of the inflammatory markers (TNF-α, IL-1β), but also augmented the expression of TGF-β1, a biomarker of immune disorder. In addition, sepsis led to injury of the kidney proximal tubules, indicated by increased KIM-1 mRNA expression (*P* < *0*.*05*). In contrast, the gingerols diminished the sepsis-induced increase in the transcription of these biomarkers (Fig. 2a–d).

Qualitative histopathological analyses indicated a few alterations in the renal tissue of septic animals, such as renal epithelial injury, inflammatory cellular infiltration and vacuolization. However, they were characterized as mild and were slightly reduced by the gingerol compounds. In addition, these findings were not quantitatively differentiated between groups (Supplementary Fig. S1). The morphological assessment revealed that both gingerols (6G and 10G) did not modify the renal morphological aspects of sham-operated animals. Sepsis (faecal peritonitis) was confirmed in CLP animals by the increased number of colony-forming units in exudate and blood bacterial load compared with the sham groups (*P* < *0*.*05*) (Supplementary Fig. 2a,b).

### Early identification of metabolic disturbance and gingerol-induced increased survival in septic AKI

**Figure 3:**
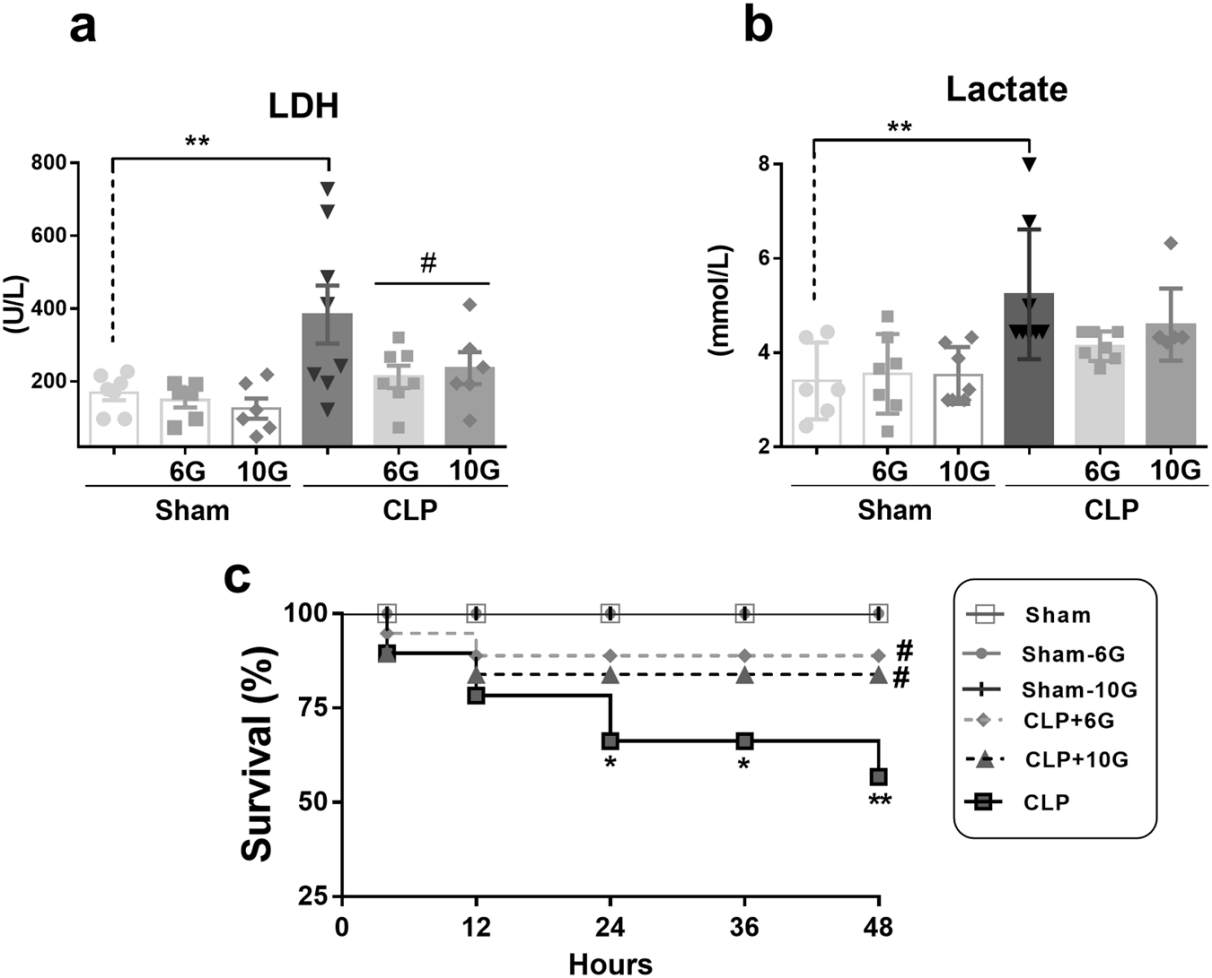
Gingerols increased survival in animals with septic AKI and decreased cell disorders. (**a**) LDH activity and (**b)** Lactate were used to verify the worsening of cellular functioning, denoting cellular disturbance in rats with septic AKI. Gingerol intervention ameliorated theses physiological responses. (**c)** Animals treated with both phenolic compounds, 6G and 10G 25 mg/Kg, preserve survival in septic AKI. The sham, sham-6G and sham-10G groups consisted of 8 animals and showed a 100% survival rate. The CLP group started with 15 animals, but only 8 animals survived. On the other hand, the CLP + 6G and CLP + 10G groups started with 11 animals, with the survival of 8 animals. The results are expressed as means ± SEM. Statistical analysis was performed by ANOVA followed by Bonferroni’s multiple comparison test. **and *denote statistical significance compared to the sham group (*P* < *0*.*01*, *P* < *0*.*05*, respectively); ^#^and ^##^show significant difference compared to CLP group (*P* < *0*.*01*, *P* < *0*.*05*, respectively).

CLP animals showed cell disruption caused by increased activity of serum lactate dehydrogenase (LDH) and higher levels of serum lactate. The gingerols decreased the LDH activity; however, the respective decrease in serum lactate levels was not statistically significant (Fig. 3a,b). In animals subjected to the CLP procedure, the survival rate decreased to 62.24% and 56.76% at 24 and 48 h, respectively. However, 6G and 10G improved the survival rate to 88.81% and 83.88%, respectively. All animals in the sham groups survived for 48 h after false surgery (Fig. 3c).

### ^1^H nuclear magnetic resonance (NMR) spectral profile of urine samples

**Figure 4:**
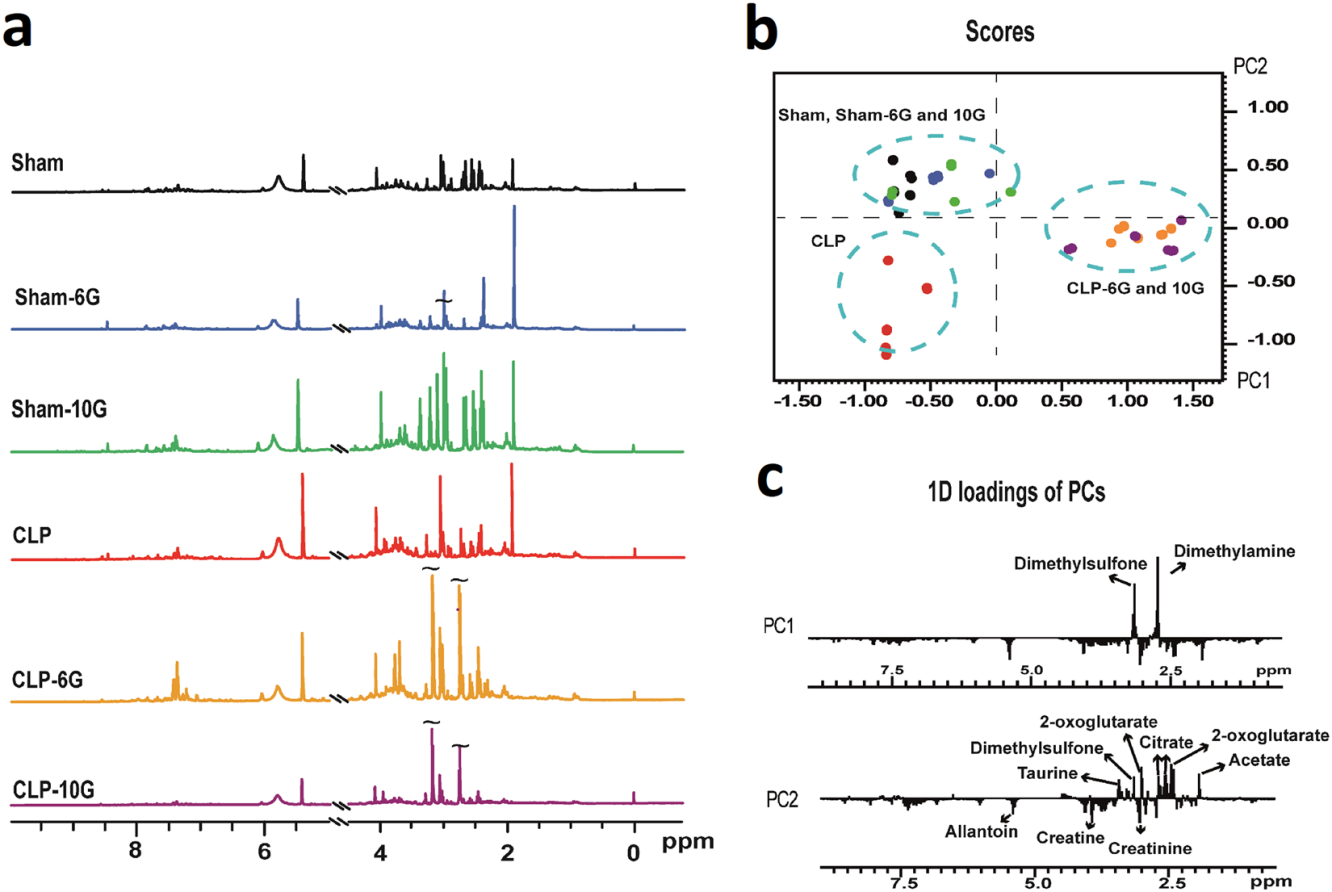
Characterization of metabolomic profile on sepsis-induced AKI. **(a)** Representative ^1^H NMR spectral profile of rat urines from animals belonging to the control group (sham), 6G group control (sham-6G), 10G group control (sham-10G), CLP-induced group (CLP), and gingerol-treated groups (CLP-6G and CLP-10G). (**b**,**c)** The PCA scores and the loadings from ^1^H NMR spectra of urine samples revealed the natural grouping of samples and the metabolites responsible for the events.

Visual inspection of the ^1^H NMR spectra of urine revealed differences in the composition of metabolites (Fig. 4a). Scores and loading plots obtained through principal component analysis applied to the bucketed spectra of urine are depicted in Fig. 4b,c.

PC1 and PC2 together explained 57.31% of the total variance. Individually, PC1 represented 45.34% and PC2 described 11.97%. The plotted scores showed a well-defined discrimination of the three groups. The sham, sham-6G, and sham-10G groups occupied the negative side of PC1 and the positive side of PC2. The CLP group occupied the negative regions of PC1 and PC2. Finally, the CLP-6G and CLP-10G groups occupied the positive region of PC1 and were dispersed along PC2 (Fig. 4b,c). The compounds with the highest impact on data variance and their respective buckets are shown in Supplementary Table S1.

**Figure 5:**
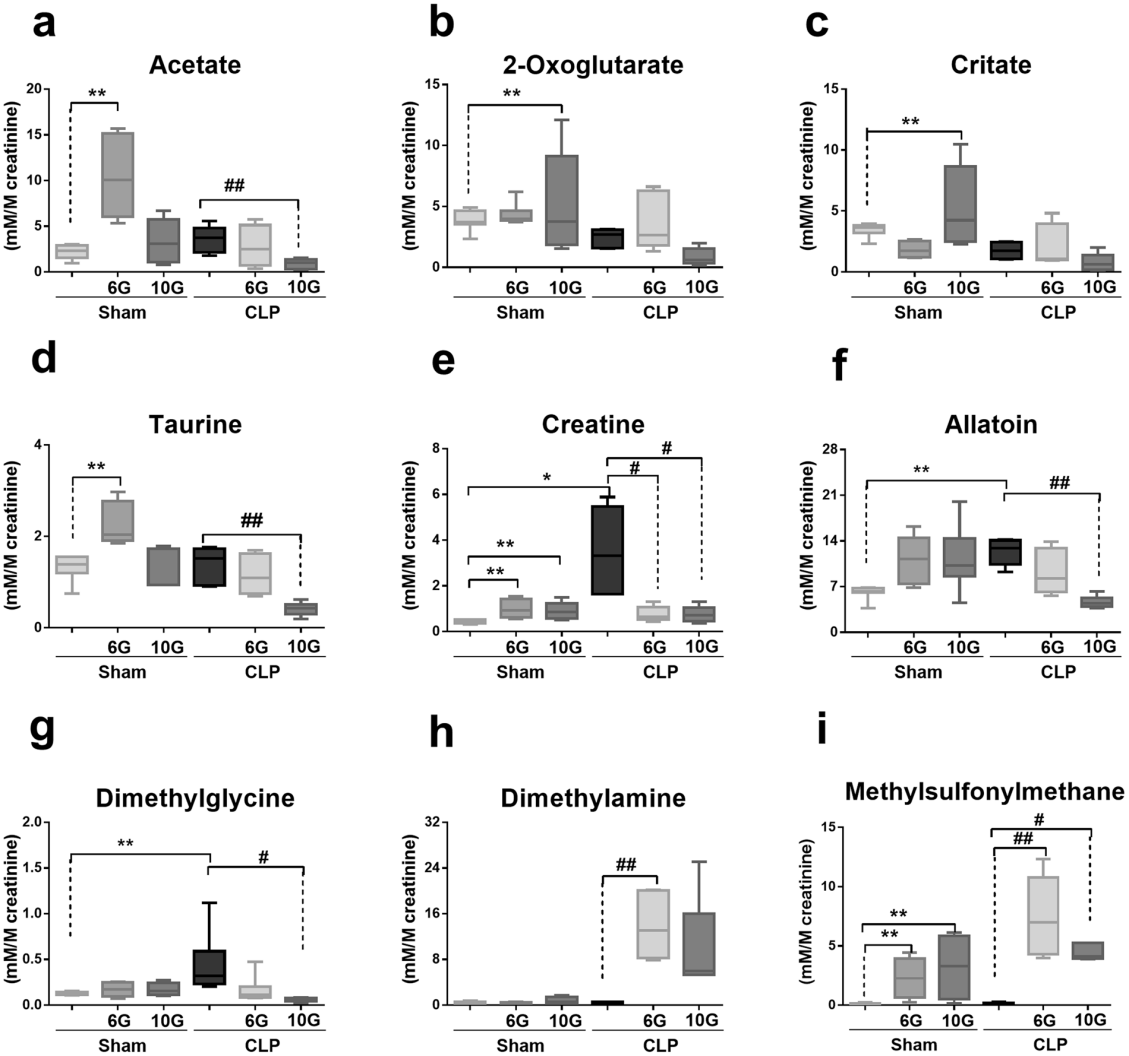
The concentration of individual metabolites (expressed as mM/M of creatinine). The results are expressed as median (n = 7–8). Quantification of the major metabolites in the urine of the experimental groups: (**a**) acetate, (**b**) 2-oxoglutarate, (**c**) citrate, (**d**) taurine, (**e**) creatine, (**f**) allantoin, (**g**) dimethyglycine, (**h**) dimethylamine and (**i**) methylsulfonylmetane. Statistical analysis was performed by Kruskal-Wallis followed by Dunn’s test. **and * denote statistical significance compared to the sham group (*P* < *0*.*05*, *P* < *0*.*01*, respectively); ^#^and ^##^show significant difference compared to CLP group (*P* < *0*.*01*, *P* < *0*.*05*, respectively).

According to the loading plot, the buckets related to 2-oxoglutarate, acetate, citrate, methylsulfonylmethane (MSM), and taurine compounds were responsible for the clustering of the sham-6G and sham-10G groups. Creatine, dimethylglycine (DMG), and allantoin influenced the discrimination of CLP samples. Taking into account all the CLP-6G and CLP-10G samples, the buckets associated with the signals of dimethylamine (DMA) and MSM were responsible for their discrimination (Fig. 5a–i and Supplementary Table S1). Owing to the relevance of gingerol compounds in the presented phenomenon, their content was determined using NMR (Supplementary Table S2).

## Discussion

In this study, septic animals demonstrated significant oliguria, disruption of tubular function, and increased urinary osmolality. However, the kidney tissue of animals with induced sepsis did not present critical morphological changes. These findings are similar to those found in other preclinical sepsis models^4,19^. Moreover, the GFR was significantly reduced and the animals showed considerable azotaemia, increased urinary protein levels, and metabolic abnormalities. AKI remains a complex disease; thus, the combination of KIM-1 and other biomarkers reflected the different stages and is specific to each diagnosis. The higher expression of KIM-1 in association with decreased levels of renal function indices (CrCl, SCr, BUN, and FE_NA_) confirms tubular deterioration^20,21^.

The septic AKI animals reported here showed significantly increased NOx content after the sepsis process. Cell damage mediated by NO can be directly correlated with peroxynitrite formation, an important nitrogen species that acts as a pathogenic agent in the peritubular capillaries in the glomerular and tubular epithelium^3,22^. ROS and RNS productions were linked to decreased antioxidant sources, specifically GSH and SOD. GSH and SOD are relevant antioxidants in renal cells that are responsible for preventing cell component damage trigged by free radicals^23^.

The present model indicated the involvement of inflammatory response in the pathogenesis of AKI, as confirmed by increased renal tissue mRNA expression of TNF-α, IL-1β, and TGF-β1. These molecules play significant roles in the pathogenesis of sepsis, which is correlated with multiple organ failure, immunosuppression, and increased mortality rates^24–26^.

The 6G and 10G treatments exhibited renoprotective effects against sepsis-induced AKI, indicated by an increase in GFR, followed by decreases in the SCr and BUN levels and urinary protein overload. These ginger-derived substances also reduced the urinary protein loss related to diabetic nephropathy and acute nephrotoxicity^16,17^, and improved the drug-induced tubular dysfunction and morphological changes related to ATN and AKI. These mechanisms are attributed to the scavenging of free radicals and the repression of NO metabolism in the renal epithelium^15,18^.

Gingerol treatment attenuated the damage caused by ROS/RNS in the renal tissues of septic rats, neutralizing the aggressive effects of the reactive species. The antioxidant effects were evidenced by the decrease of malondialdehyde and increase of GSH activity. Our group previously showed the antioxidant effects of gingerols (6G and 10G) against ATN^15^. Recent reports have also indicated that 6G inhibits the death of tubular and glomerular cells^27^. In addition to the antioxidant effects, ginger compounds decrease the up-regulation of cytokine expression in kidney tissue. Thus, in studies with human cells, 6G decreased the production IL-1β and IL-8 and suppressed nuclear factor-κB (NF-κB)^15,28^.

In the present study, renal failure occurred with metabolic collapse in septic animals, which was evidenced by hyperlactataemia, an indication of poor tissue oxygenation^29^. Increased LDH activity is also indicative of cell metabolism impairment, which is frequently used to monitor sepsis-induced renal injury^22,30^ and often noted in patients with sepsis^8,30,31^.

The pathogenesis of sepsis is frequently related to multiple organ dysfunction^10,32^. A multivariate analysis revealed that metabolic disorder was consistently correlated with nitrogen metabolism in the septic AKI group, as evidenced by increased creatine, allantoin, and DMG levels. Septic stress induced by a polymicrobial infection is known to cause metabolic perturbations with a possible disruption of protein and energy metabolism^30^. The catabolic rates of patients with sepsis are significantly higher than their anabolic rates, and this difference results from neuroendocrine responses that are closely related to the activities of cytokines and some inflammatory mediators. Thus, critical fluctuations in the levels of amino acids in patients with sepsis and those with SIRS were recently revealed. The serum taurine concentrations were lower as the severity of septic disease increased within 14 days^33^. In the present study, the taurine concentrations did not change in the CLP group in contrast to other preclinical studies^11,32^, and this result is probably related to the characteristics of the present model. However, higher creatine concentrations aid in the identification of disruptions in energy protein metabolism^11,32^.

Allantoin, an end-stage degradation product of purine catabolism, is produced via the peroxidation of uric acid and protein degradation. A recent proteomics study showed that an increased allantoin concentration was associated with decreased renal function in patients with SIRS^34^.

The present findings indicate that the gingerols modulated fatty acid and protein metabolism in addition to disrupting the glycolytic pathway. The elevated levels of acetate, 2-oxoglutarate, and creatine metabolites noted in the sham groups suggested the ability of gingerols to affect some important catabolic pathways. However, this issue remains to be elucidated. On the contrary, in the CLP-operated animals, 6G and 10G treatments decreased the amounts of urinary creatine and allantoin produced by septic animals, which could indicate a normalization of cellular function after the induction of septic AKI. The increase in the levels of taurine, an amino acid with important antioxidant actions^33,35^, through the 6G treatment can indicate the beneficial mechanisms attributed to this gingerol.

^1^H NMR analysis revealed higher urinary excretion of DMA and MSM after the intraperitoneal (ip) administration of the gingerol compounds, especially in the CLP-treated animals. These metabolites have been related to inflammatory processes, particularly in the pathogenesis of systemic infection^36^. A higher serum DMA level was detected in non-survivors than in survivors of septic shock, which could be an indicator of the severity associated with the severity of sepsis injury^37^. MSM, a sulphur-containing organic compound that occurs naturally in the urine of humans and other mammals, is a metabolite with beneficial action in inflammatory diseases such as arthritis, allergies, and certain parasitic infections^38^. MSM has proven anti-inflammatory and antioxidant properties *in vitro*. It was also associated with the decreased production of oxidative compounds, including hydrogen peroxide, superoxide, and hypochlorous acid, by human neutrophils^39^. MSM treatment also suppressed the NF-κB pathway and decreased the levels of NO after lipopolysaccharide-induced kidney damage^38^. Together, these previous data corroborate the present findings.

In addition, it has been demonstrated that MSM has protective mechanisms against the metabolic syndrome triggered by hyperglycaemia, modulating enzymatic actions and its biochemical pathways, as well as reducing the inflammatory cytokines. Moreover, as in inflammasome activation in mouse and human macrophages, MSM significantly attenuates the transcriptional expression of IL-1α, IL-1β, IL-6, and NLRP3 (NLR family pyrin domain-containing 3), confirming its anti-inflammatory characteristics^40,41^. In the present study, we hypothesized that a considerable amount of urinary DMA and MSM in the sepsis groups treated with gingerols may be a positive sign of the restoration of kidney function. Thus, DMA excretion could be a biomarker of the severity associated with improved renal rates and increased survival. On the other hand, the high values of urinary MSM correlated with the antioxidant and anti-inflammatory mechanisms of both treatments demonstrated in the present study. Therefore, treatment with gingerols could putatively modulate inflammatory and oxidative diseases via pathways related to MSM^38,39^.

**Figure 6:**
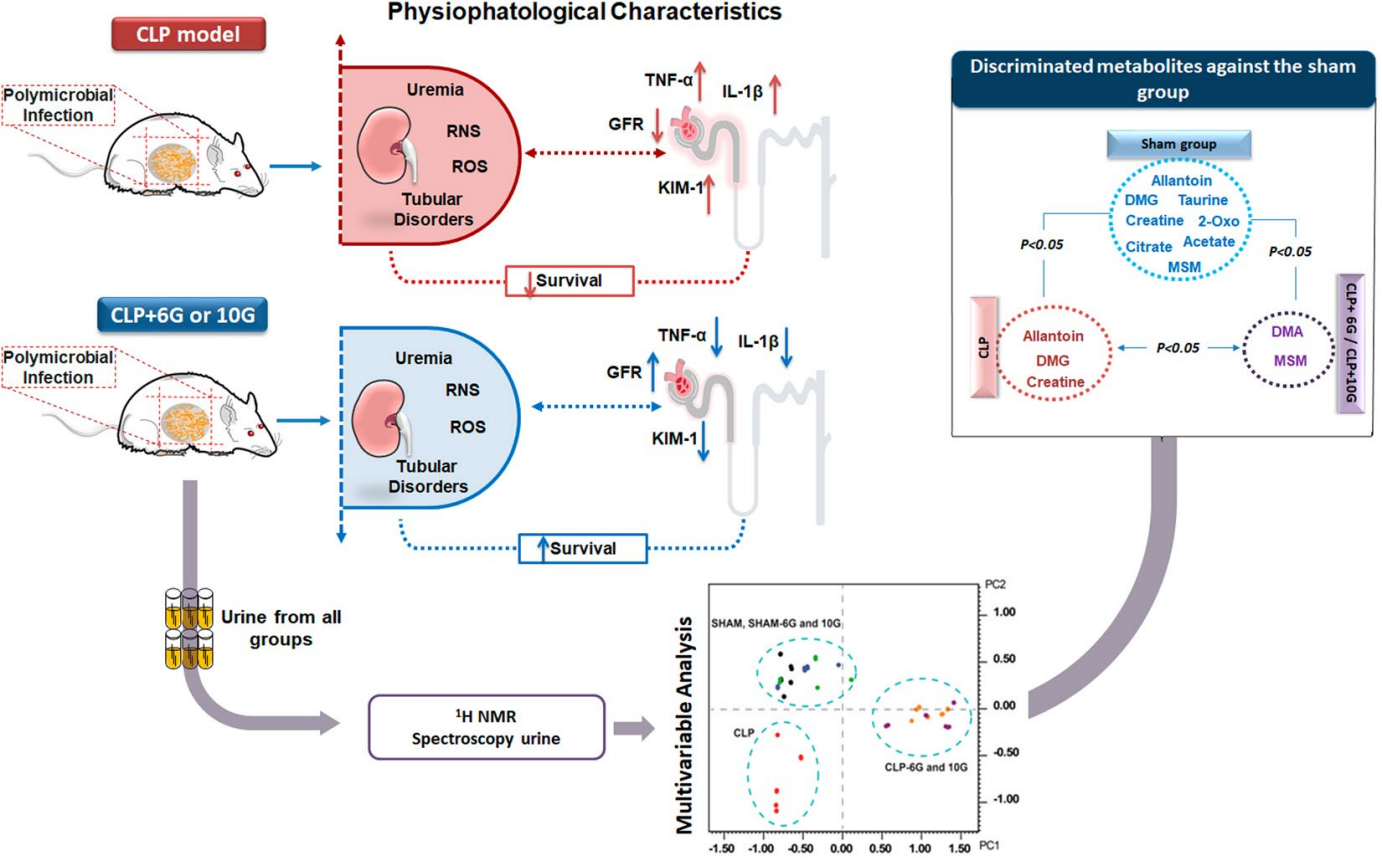
Summary of the key findings related to Gingerols activity on septic AKI and metabolic description. There are interesting protective effects of the phenol compounds with indication for intervention of renal injury. Both experimental procedures induced three different metabolomic profiles.

In conclusion, we showed, for the first time, the beneficial effects of ginger phenolic compounds against septic AKI, through their antioxidant and anti-inflammatory properties (Fig. 6). It is also conceivable, via the metabolomic approach, that urinary DMA and MSM metabolites could be used as biomarkers in septic AKI treatment.

## Materials and Methods

### *In vivo* experiments

Male Wistar rats (7 weeks old, weighing 180–210 g), supplied by Center Vivarium at the Federal University of Ceará (Fortaleza, Brazil), were used. All experimental protocols were conducted in accordance with the principles for the humane use of animals for scientific research purposes of the CONCEA (National Council for Control of Animal Experimentation), and approved by the Ethical Committee for Animal Use of the Federal University of Ceará, Fortaleza, Brazil (protocol 45/14). The 6G and 10G compounds were isolated as previously described by Silva *et al*.^42^. Septic AKI was induced by using the CLP model, as previously described^4,43^. After a midline laparotomy, the caecum was exposed and a 3–0 silk ligature was placed at 0.80 cm of the caecal tip, and punctured five times with an 18-gauge needle. The caecum was gently squeezed until a 1-mm column of faecal material was exteriorized. The caecum was returned to the abdominal cavity, and the incision was closed in layers. Lastly, to ensure adequate fluid therapy, each animal was administered 0.15 M NaCl (25 ml/kg, ip). The animals of the sham groups were subjected to false surgery, without caecal ligation and puncture.

### Experimental design and groups

The animals (N = 61) were randomly assigned to their respective groups (n = 8–15). Treatments were performed 2 h before as well as 12 and 24 h after the CLP surgical procedure^15^, as demonstrated in Supplementary Fig. S3. The animals were divided into the following groups: 1) sham, subjected to false surgery and treated with vehicle (2.5% Tween 80 ip); 2) sham-6G, subjected to false surgery and treated with 6G 25 mg/kg ip; 3) sham-10G, subjected to false surgery and treated with 10G 25 mg/kg ip^17,18^; 4) CLP, subjected to the CLP surgery and treated with vehicle (2.5% Tween 80 ip); 5) CLP + 6G, subjected to CLP surgery and injected with 6G 25 mg/kg ip; and 6) CLP + 10G, subjected to CLP surgery and injected with 10G 25 mg/kg ip. After all procedures, the number of animals per group (n = 7–8) depended on the survival rates.

### Biochemical and morphological analyses and bacterial counting

Blood and urine samples collected from animals in metabolic cages were used to measure the BUN, SCr, urinary protein, LDH, and lactate levels (Labtest, Fortaleza, Brazil). Plasmatic and urinary sodium (Na^+^) concentrations were determined using the selective ion method (AVL 9180 Electrolyte Analyser®; Roche, Brazil) and used as indicators of renal tubular function through the estimation of sodium excretion (FE_NA_). Urine osmolality was determined using a vapour pressure osmometre (model 5520; Wescor, Logan, UT, USA). Peritoneal lavage fluid and blood samples were also collected. These samples were diluted carefully and plated into Mueller-Hinton agar dishes (Mueller Hinton Agar; Difco, France). After 24 h of incubation at 37 °C, bacterial concentrations were determined by counting the colony-forming units. The kidneys were stained with haematoxylin and eosin. Light microscopic sections were assessed for the presence of tubular cell necrosis, inflammatory cell infiltration, loss of brush border, tubular dilation, cast formation, and cellular oedema in the tubular interstitium^44,45^. The findings were expressed by using a semi-quantitative scale according to the percentage of damaged tubules: 0, no damage; 1, <25% damage; 2, 25–50% damage; 3, 50–75% damage, 4, 75–90% damage; and 5, >90% damage.

### Assessment of oxidative and nitrosative stress in the kidney

Malondialdehyde was assayed in renal tissue samples by measuring thiobarbituric acid–reactive substances. Nitrite level was determined using Griess reagent based on a colorimetric assay^46^. SOD activity and GSH level were assayed as previously described^47,48^.

### Kidney inflammatory status

The mRNA expression of TNF-α, IL-1β, TGF-β1, and KIM-1 was evaluated using reverse transcription–quantitative polymerase chain reactions. Glyceraldehyde 3-phosphate dehydrogenase (*GAPDH*) was used as a housekeeping gene. The DNA primers for all genes are described in Table 2. Gene expression analysis was performed using the mathematical 2^∆∆CT^ method^49^.

**Table 2:** Oligonucleotide sequences of primers used for RT-qPCR.

Gene	Primer direction	Primer sequence (5′-3′)
TNF-α	Sense	GTACCCACTCGTAGCAAAC
	Antisense	AGTTGGTTGTCTTTGAGATCCATG
IL-1β	Sense	GACCTGTTCTTTGAGGCTGACA
	Antisense	CTCATCTGGACAGCCCAAGTC
TGF- β1	Sense	GGGCTACCATGCCAACTTCTG
	Antisense	GAGGGCAAGGACCTTGCTGTA
KIM-1	Sense	CGCAGAGAAACCCGACTAAG
	Antisense	CAAAGCTCAGAGAGCCCATC

### Acquisition, assignment, and processing of NMR data

All NMR spectra were recorded at 300 K using an Avance DRX-500 spectrometer operating at a ^1^H frequency of 499.6 MHz, equipped with a 5-mm broadband inverse probe. ^1^H NMR spectra were acquired through a water suppression pulse sequence, noesypr1d (Bruker library), using 64 K data points over a 25-ppm spectral width averaged over 128 transients. Free induction decays were Fourier transformed with line broadening of 0.3 Hz. The resulting spectra were manually phased, baseline corrected, and referenced to the 3-(trimethylsilyl) propionate resonance (methyl groups) at 0.0 ppm. Compounds present in urine samples were identified using one-dimensional and two-dimensional NMR experiments, as well as through comparisons of the obtained data with the NMR data in open access databases and literature reports^50,51^. Principal component analysis was performed on the ^1^H NMR data set by using the AMIX Statistics package (version 3.9.12; Bruker BioSpin, Rheinstetten, Germany). The metabolites responsible for the discrimination of urine samples were quantified using the original spectra data through an internal method with 3-trimethylsilyl-propionic acid sodium salt (1 mM) as the internal reference. The present methods are in accordance with previous works^52–54^.

### Statistical analysis

The multi-integration toolkit of the AMIX Viewer (Bruker BioSpin) was employed to normalize the NMR data. One-way analysis of variance followed by Bonferroni’s multiple comparison or the Kruskal-Wallis followed by Dunn’s test was used for parametric or non-parametric data, respectively. Gene expression data were evaluated using the Mann-Whitney test. *P* values < 0.05 were considered significant.

## Electronic supplementary material

Dataset 1.
