# Supplementary material for: Gingerol suppresses sepsis-induced acute kidney injury by modulating methylsulfonylmethane and dimethylamine production
Source: Sci Rep. 2018 Aug 14;8:12154. doi: 10.1038/s41598-018-30522-6 (PMC6092401; doi:10.1038/s41598-018-30522-6)
Supplement: Supplementary file 1 — Dataset 1. [file 41598_2018_30522_MOESM1_ESM.doc]

**Supplementary Contend**

**Gingerol suppresses sepsis-induced acute kidney injury by modulating methylsulfonylmethane and dimethylamine production**

Francisco Adelvane de Paulo Rodrigues, PhD^1^; Alan Diego da Conceição Santos, PhD^2^; Pedro Henrique Quintela Soares de Medeiros, MS^1^; Mara de Moura Gondim Prata, MS^1^; Tailane Caína de Souza Santos^3^; James Almada da Silva, PhD^3^; Gerly Anne de Castro Brito, MD, PhD^1^; Armênio Aguiar dos Santos, MD, PhD^1^; Edilberto Rocha Silveira, PhD^2^; Aldo Ângelo Moreira Lima, MD, PhD^1^, Alexandre Havt, PhD^1*^

**Supplementary Table S1-** ^1^H and ^13^C NMR peak assignments for identified metabolites in rat urine.

| **Metabolite** | **Buckets**  **(ppm)** | | **Assignment** | **δ^1^H** | **Multiplicity (Hz)** | **δ^13^C** |  |
| --- | --- | --- | --- | --- | --- | --- | --- |
| 2-Oxoglutarate | | 3.01 | CH_2_ | 3.01 | t (7.0) | 38.8 |  |
|  | | 2.45 | CH_2_ | 2.44 | t (7.0) | 33.5 |  |
| Acetate | | 1.93 | CH_3_ | 1.93 | S | 24.8 |  |
| Allantion | | 5.41 | CH | 5.41 | S | 65.9 |  |
| Citrate | | 2.68-2.71 | 1/2γCH_2_ | 2.68 | d (15.9) | 47.8 |  |
|  | | 2.53-2.59 | 1/2γCH_2_ | 2.55 | d (15.9) | 47.8 |  |
| Creatine | | 3.93 | CH_2_ | 3.93 | S | 56.6 |  |
|  | | 3.03 | CH_3_ | 3.04 | S | 39.9 |  |
| Creatinine | | 4.05 | CH_2_ | 4.05 | S | 59.1 |  |
|  | | 3.05 | CH_3_ | 3.05 | S | 32.9 |  |
| Dimethylamine | | 2.73 | N-(CH_3_)_2_ | 2.73 | S | 41.5 |  |
| Dimethylsulfone | | 3.15 | 2xCH_3_ | 3.16 | S | 44.4 |  |
| Dimethylglycine | | ~ | CH_2_ | 3.73 | S | ~ |  |
|  | | 2.93 | N-(CH_3_)_2_ | 2.93 | S | ~ |  |
| Taurine | | 3.43 | CH_2_ | 3.43 | t (6.52) | ~ |  |
|  | | ~ | CH_2_ | 3.27 | t (6.42) | ~ |  |

| **Position** | **6-Gingerol** | |  | **10-Gingerol** | |  |
| --- | --- | --- | --- | --- | --- | --- |
|  | δ_H_, Mult.  (J in Hz) | δ_C_ | δ_C_ Literature* | δ_H_, Mult.  (J in Hz) | δ_C_ | δ_C_  Literature* |
| 1’ |  | 134.0 | 134.2 |  | 134.1 | 134.2 |
| 2’ | 6.75 d (1.7) | 113.1 | 113.3 | 6.76 d (1.7) | 113.2 | 113.3 |
| 3’ |  | 148.8 | 149.0 |  | 148.9 | 149.0 |
| 4’ |  | 145.7 | 145.9 |  | 145.8 | 145.9 |
| 5’ | 6.68 d (7.9) | 116.1 | 116.3 | 6.68 d (8.0) | 116.2 | 116.3 |
| 6’ | 6.60 dd (7.9; 1.7) | 121.7 | 121.8 | 6.60 dd (8.0; 1.7) | 121.7 | 121.9 |
| 1 | 2.70-2.80 brd  2.73-2.83 brd | 30.2 | 30.5 | 2.73-2.83 brd  2.73-2.83 brd | 30.3 | 30.6 |
| 2 |  | 46.4 | 46.7 |  | 46.4 | 46.7 |
| 3 |  | 212.0 | 212.0 |  | 212.1 | 212.1 |
| 4 | 2.48 dd (15.8; 4.5) | 51.3 | 51.6 | 2.48 dd (15.8; 4.5) | 51.3 | 51.6 |
|  | 2.54 dd (15.8; 8.2) | 51.3 | 51.6 | 2.54 dd (15.8; 8.0) | 51.3 | 51.6 |
| 5 | 3.99 m | 68.9 | 69.2 | 3.99 m | 68.9 | 69.2 |
| 6 | 1.40 m | 38.3 | 38.7 | 1.39 m | 38.4 | 38.7 |
| 7 | 1.40 m | 26.3 | 33.2** | 1.39 m | 26.6 | 33.4** |
| 8 | 1.29 m | 32.9 | 26.6** | 1.29 m | 30.7 | 31.1 |
| 9 | 1.29 m | 23.7 | 24.0 | 1.29 m | 30.7 | 31.0 |
| 10 | 0.89 t (7.0) |  | 14.7 | 1.29 m | 30.7 | 30.0 |
| 11 |  |  |  | 1.29 m | 30.4 | 30.8 |
| 12 |  |  |  | 1.29 m | 33.1 | 26.7** |
| 13 |  |  |  | 1.29 m | 23.7 | 24.1 |
| 14 |  | 14.46 |  | 0.89 t (7.0) | 14.4 | 14.8 |
| 1’’ |  | 56.3 | 56.6 | 3.81 s | 56.4 | 56.6 |

**Supplementary Table S2 -** δ_H_ (499.6 Hz) and δ_C_ (124.9 Hz) NMR spectra data of 6- and 10-gingerol (CD_3_OD, δ in ppm and J in Hz). The table also shows ^13^C NMR data from literature.

* Kim et al. (2008).

** Although the carbon values are in line with the results presented in the present work, a few signal assignments seem to be interchanged.

**Reference**

1. Kim, J. S.; Lee, S. I; Park, H. W et al. Cytotoxic from the died rhizomes of *Zingiber officinale* Roscoe. *Arch Pharm Res* 2008; 31:415-418.

**Supplementary Figure S1**


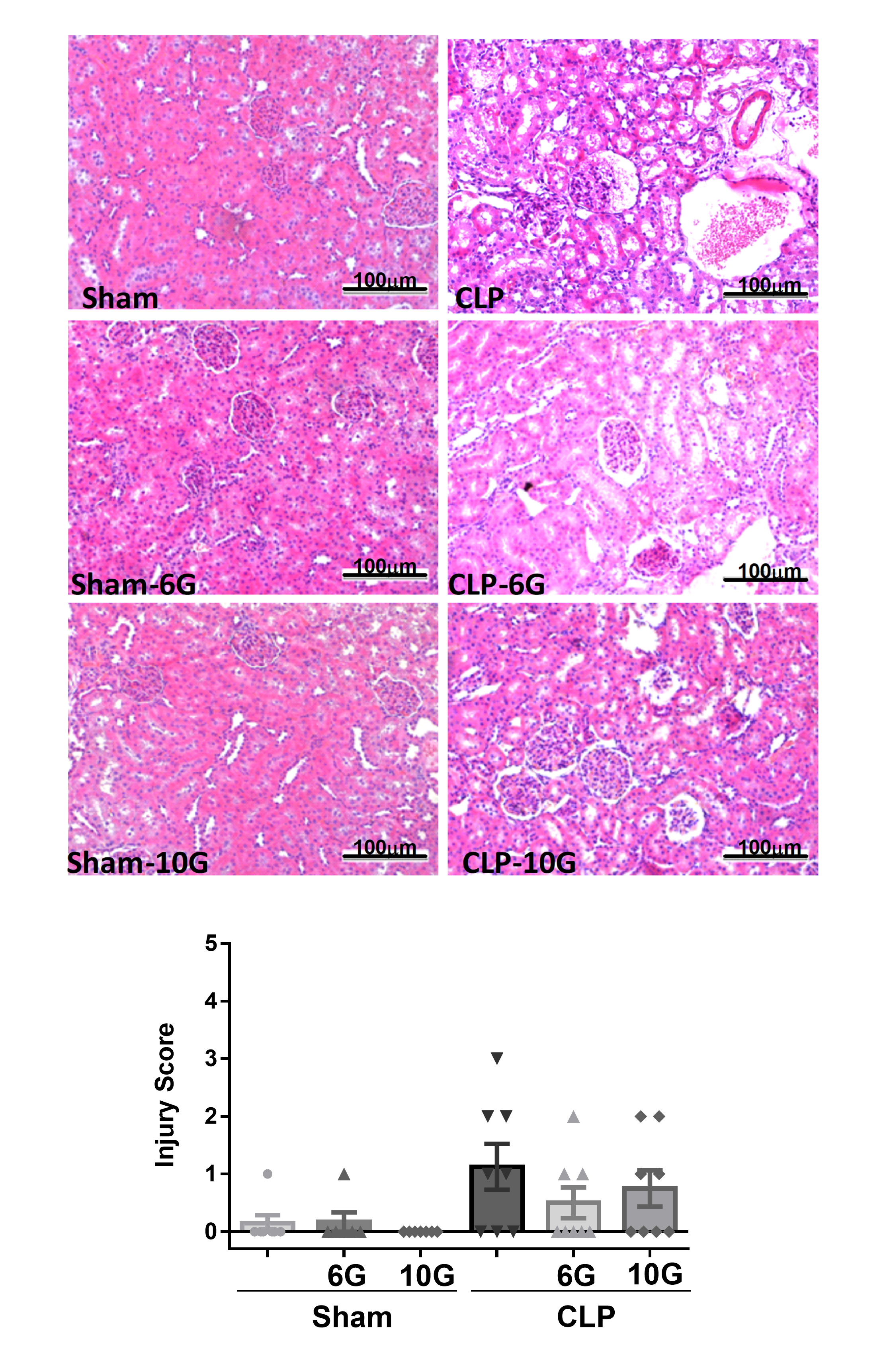


**Supplementary Figure S1-** Photomicrographs depicting H&E-stained sections of kidneys from experimental groups. a) The sham group, sham-6G, sham-10G, had no indications of kidney cell alterations. There was vacuolar degeneration in proximal tubule cells and inflammatory cells infiltration in CLP group associated with CLP-induced sepsis. These features were attenuated by subsequently 6-gingerol and 10-gingerol treatment (CLP+6G and CLP+10G). Magnification 400x. Statistical analysis was performed by Kruskal-Wallis followed by Dunn’s test.

**Supplementary Figure S2**


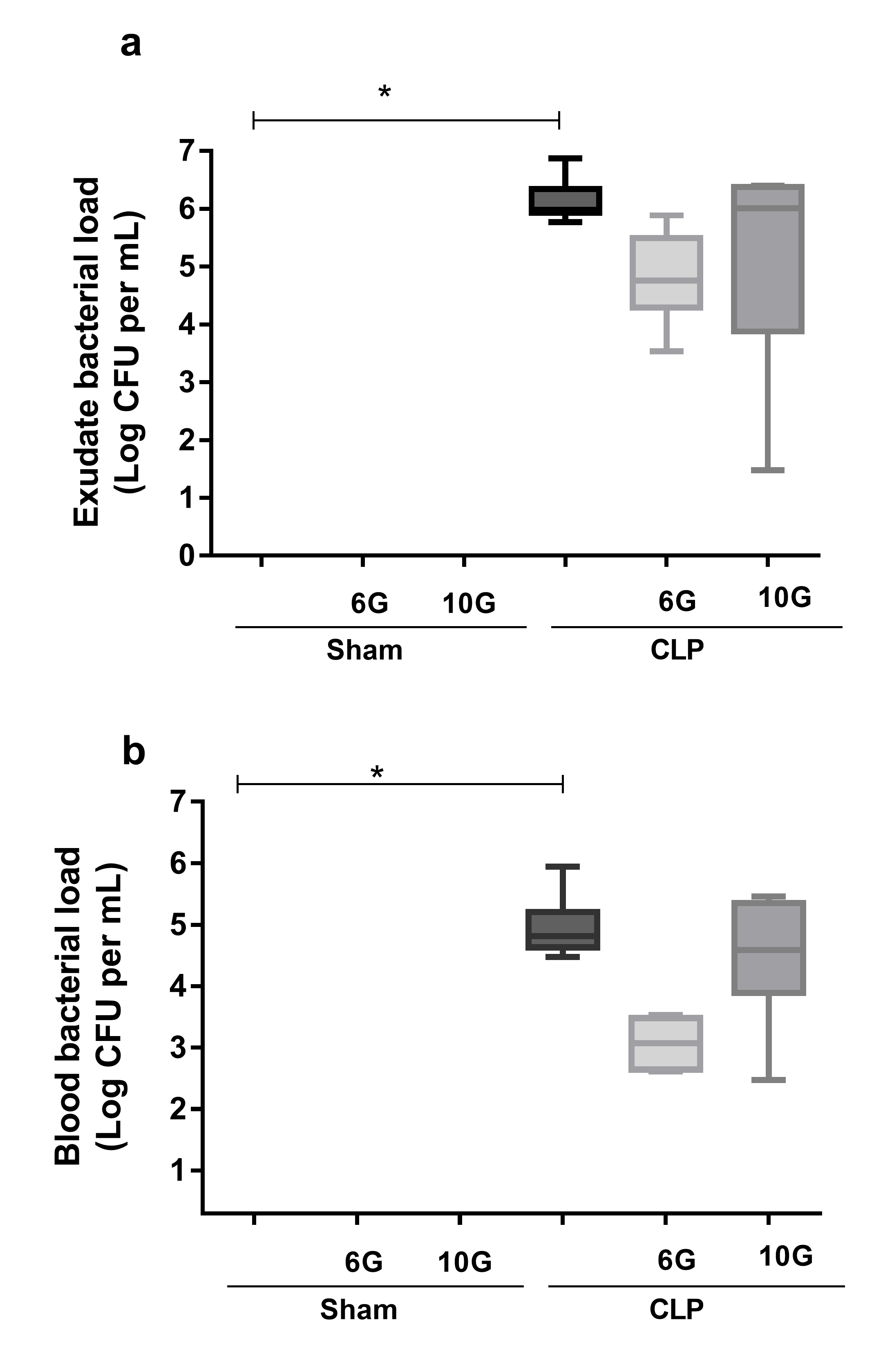


**Supplementary Figure S2-** CLP process induces infection in animals with significant bacterial growth. Statistical analysis was performed by Kruskal-Wallis followed by Dunn’s test. *** and *** denote statistical significance compared to the sham group (*P<0.05, P<0.01,* respectively).

**Supplementary Figure S3.**


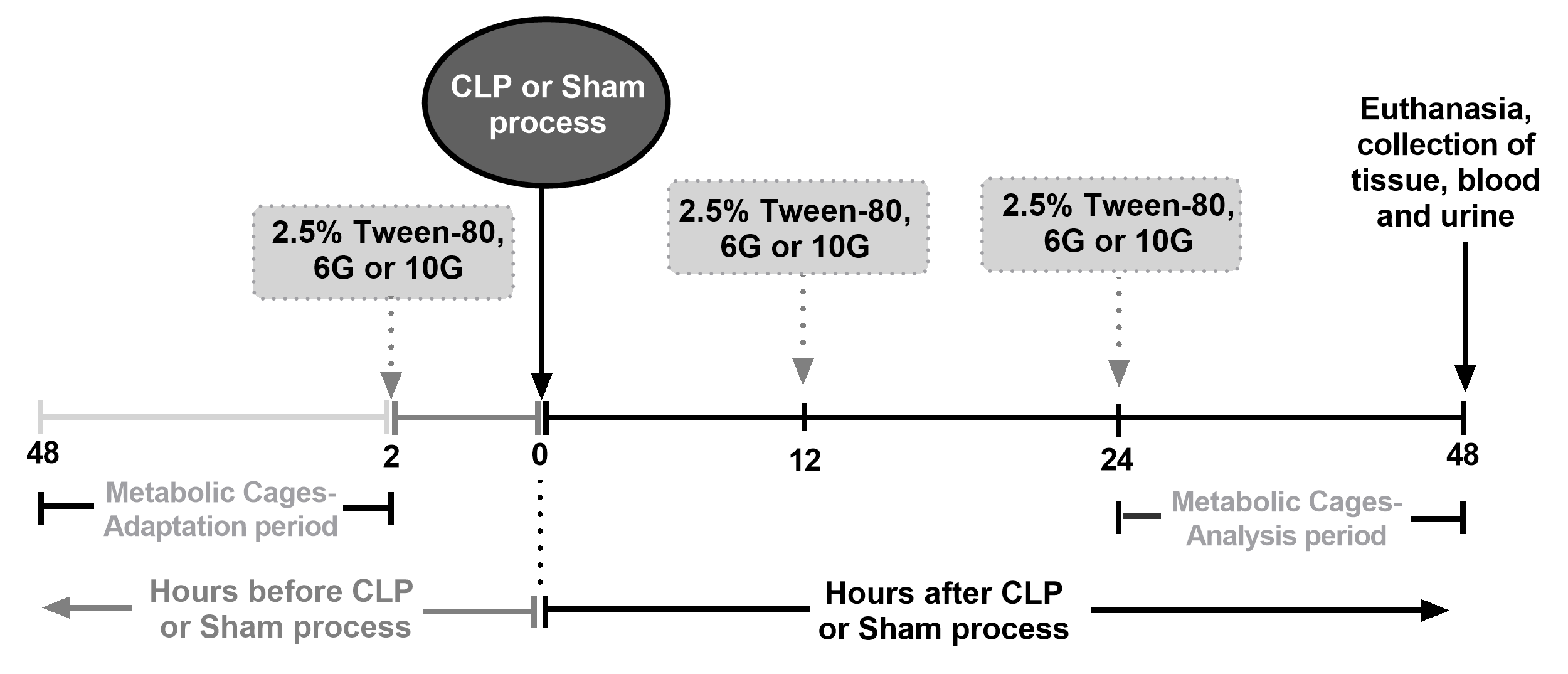


**Supplementary Figure** **S3**- **Schematic representation of experimental design**. The present protocol consisted of a 2-hour pre-treatment with tween, 6G or 10G compounds, followed by CLP or Sham procedures. After, 12 and 24 hours of surgery procedures, the animals were treated again with gingerols or 2.5% Tween-80. Next, the animals were housed in the metabolic cages for 24 hours when we evaluated all parameters from the collected tissues and biofluids.
